# Management of Tracheal Perforation following Anterior Cervical Spine Surgery: Report of Two Cases and Review of the Literature

**DOI:** 10.1155/2022/1914642

**Published:** 2022-04-05

**Authors:** Xinhu Guo, Hongquan Ji

**Affiliations:** ^1^Department of Orthopaedics, Peking University Third Hospital, China; ^2^Beijing Key Laboratory of Spinal Disease Research, China

## Abstract

**Background:**

Tracheal perforation is a complication very rare but challenging that follows anterior cervical spine surgery. This article describes the management of tracheal perforation due to instrument failure after anterior cervical spine surgery performed in two patients because of fracture dislocation of the subaxial cervical spine. *Case Presentation*. Two patients who suffered from a subaxial cervical fracture and dislocation were subjected to anterior cervical spine surgery for fracture reduction and cervical fusion. However, instrumentation failure occurred in both patients, resulting in implant displacement and penetration into the posterior tracheal wall. Revision surgery consisted of fracture reduction, multilevel posterior fixation, and removal of the displaced anterior cervical implants. Tracheal perforation was bypassed by placing a tracheostomy tube in a caudal position for the diversion of the airflow and tracheal hygiene. The thorough debridement and drainage performed in both patients allowed a complete healing of the anterior wound in both of them, with no sign of infection or subcutaneous emphysema, as confirmed by postoperative CT scan and flexible bronchoscopy. Both patients acquired a solid fusion of the cervical spine at last follow-up (16 months and 24 months).

**Conclusions:**

The perforation of the trachea after anterior cervical spine surgery due to the displacement of the implants could be managed using posterior cervical instrumentation and fusion, the removal of the anterior implant, debridement and drainage, and the use of a distal bypassing tracheostomy tube.

## 1. Introduction

Anterior cervical spine surgery is frequently performed on patients with cervical spondylosis, radiculopathy, myelopathy, and trauma to the cervical spine [1]. This surgery has been performed safely and effectively for decades, although some complications may occur, which include dysphagia, C5 palsy, graft or hardware failure, hematoma, and esophageal perforation [2]. Among these complications, tracheal perforation is very rare but challenging, and it can be fatal if not rapidly recognized and cured [3–5]. To date, a search of the PubMed database revealed the presence of only 2 related case reports [3, 4]. The purposes of this article were (1) to report two cases of the very rare complication of tracheal perforation following anterior cervical spine surgery and (2) to introduce our experience on the treatment of this rare complication.

## 2. Case Presentation

### 2.1. Case 1

**Figure 1:**
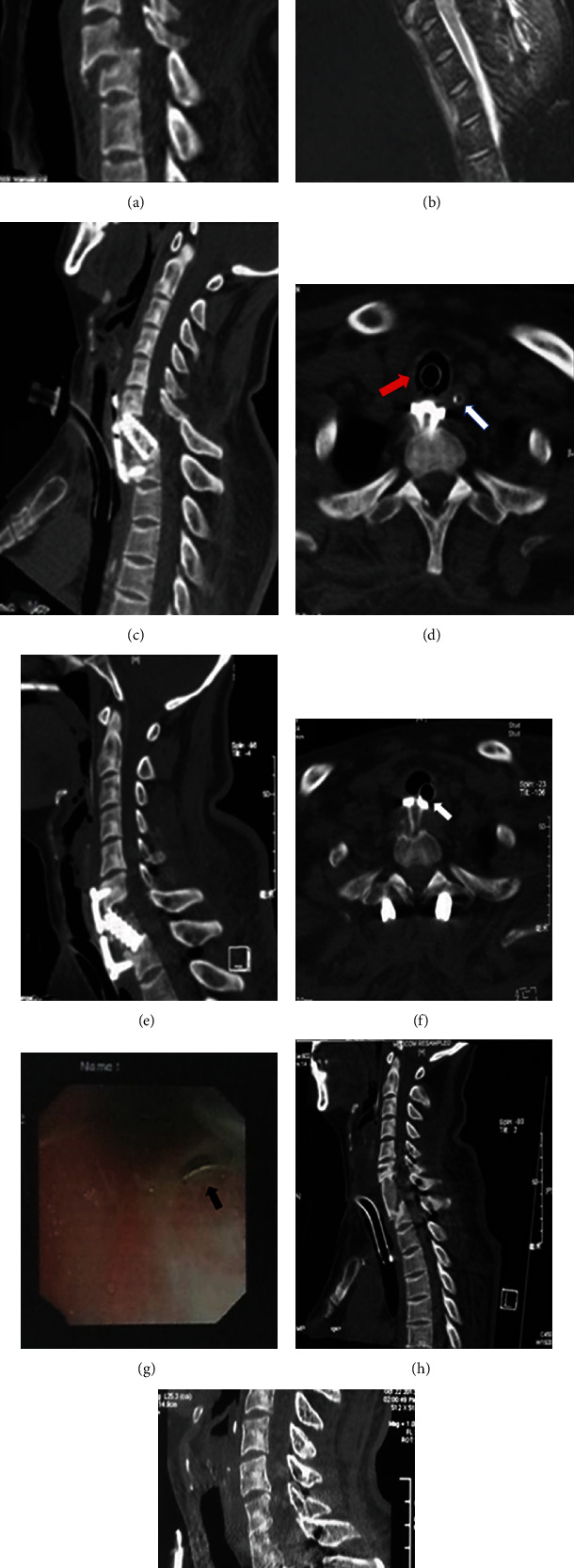
Images of case 1. (a) Preoperative sagittal CT reconstruction showing the dislocation of C6 and fracture of C7; (b) T2-weighted sagittal MR showing the disruption of the discoligamentous elements from the anterior longitudinal ligament to the nuchal ligament and spinal cord injury (as shown by the extensive high signal intensity of the spinal cord); (c) postoperative sagittal CT reconstruction revealing the dislocation of the C6 and the failure of the instrumentation; (d) postoperative axial CT scan showing the back out of the screws and the encroaching of the plate on the trachea. The white arrow indicates the esophagus, with the gastric tube inside, and the red arrow indicates the trachea, with the endotracheal tube inside; (e) after posterior revision, sagittal CT reconstruction was performed, showing the restoration of the cervical alignment, but the anterior implant failure was more significant; (f) axial CT scan after posterior revision showing the encroachment of the plate on the trachea, with the plate in contact with the endotracheal intubation (white arrow); (g) flexible bronchoscopy after posterior revision showing the perforation of the posterior tracheal wall with the screws/plate penetrating into the lumen of the trachea (black arrow); (h) sagittal CT reconstruction after the anterior revision showing the strut bone graft between C6 and T1 and a more distal tracheostomy tube; and (i) sagittal CT reconstruction showing the maintained alignment of the cervicothoracic junction and the solid fusion between C6 and T1 16 months after the anterior revision procedure.

A 54-year-old woman was involved in a car accident that caused the fracture of the C7 vertebra and the anterior dislocation of the C6 (Figure 1(a)); thus, she suffered from a complete cervical cord injury according to the American Spinal Injury Association (ASIA) impairment scale, grade A (Figure 1(b)). She underwent anterior cervical corpectomy and fusion (ACCF) of the C7 on the second day after the accident. She stayed intubated for seven days as a result of impaired respiratory function due to the complete injury of the cervical cord, and tracheotomy was performed on the post-operative day (POD) 8. CT scan on POD 18 revealed the implants displacement and loss of reduction of C6 dislocation (Figures 1(c) and 1(d)). She underwent posterior fracture reduction, fixation, and fusion on POD 35. Alignment of the cervical spine was restored, but the distal screws were still backed out and were invading the trachea along with the anteriorly displaced plate (Figures 1(e) and 1(f)). The anterior revision procedure was postponed because of recurrent pneumonia and concerns on her ability to withstand another surgery. On POD 33 (after the posterior revision procedure), a fiber-optic bronchoscopy revealed the penetration of the displaced implants into the posterior tracheal wall (Figure 1(g)). Anterior debridement, irrigation, and the removal of the implants were performed. The vertebral defect between C6 and T1 was filled with autologous bone graft harvested from her anterior iliac crest (Figure 1(h)). However, the tracheal perforation was not repaired because of the difficulty and danger in the exposure of the posterior perforation. The wound was primarily closed, a negative pressure drainage was used for the wound, and a tracheostomy tube was reinserted distal to the tracheal perforation to replace the oral endotracheal tube immediately after the anterior revision procedure. Wound healing was successful, with no signs of subcutaneous or mediastinal emphysema. On POD 30 (after the anterior revision procedure), a fiber-optic bronchoscopy was performed, showing the perforation had healed. At the time of last follow-up (16 months), CT scan revealed the presence of a solid fusion from C6 to T1, and the trachea was intact (Figure 1(i)).

### 2.2. Case 2

**Figure 2:**
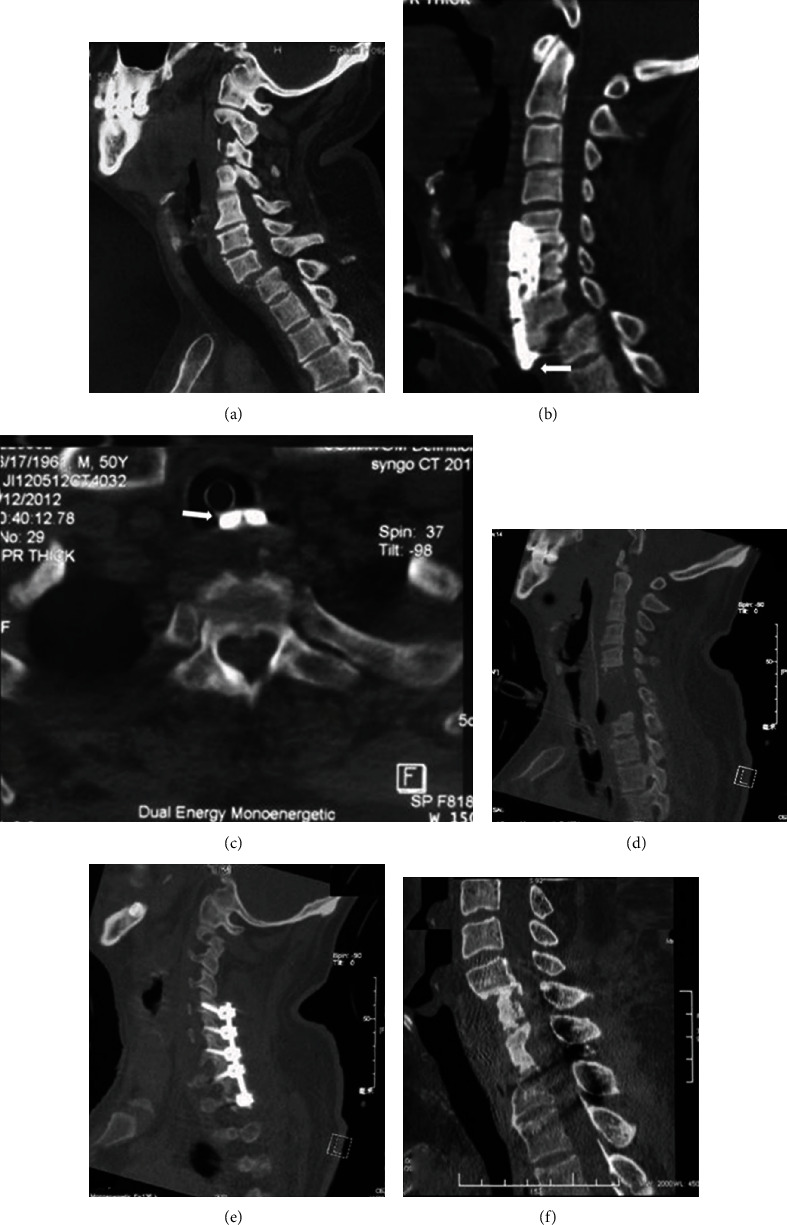
Images of case 2. (a) Preoperative sagittal CT reconstruction showing the anterior dislocation of C7; (b) postoperative sagittal CT reconstruction showing the failure of the instrumentation and the penetration of the distal end of the plate into the posterior tracheal wall (white arrow); (c) axial CT scan showing the penetration of the plate into the lumen of the trachea, with the plate in contact with the endotracheal intubation (white arrow); (d) sagittal CT reconstruction following the revision procedures demonstrating the restoration of the alignment of the cervicothoracic junction; (e) sagittal CT reconstruction following the revision procedures showing the posterior reduction and fixation from C5 to T2; and (f) sagittal CT reconstruction 24 months after the revision procedures showing the good maintenance of the alignment of the cervicothoracic junction.

A 50-year-old man fell from height, causing a C7-T1 dislocation that resulted in a complete cervical spinal cord injury (ASIA, grade A) (Figure 2(a)). He underwent C6-7 partial ACCF and tracheotomy in a local hospital. CT scan on POD 13 showed implant failure with suspected tracheal perforation (Figures 2(b) and 2(c)), and he was transferred to our department. An anterior revision procedure was done to remove the implant. A thorough debridement was performed, the vertebral defect between C5 and T1 was left unrepaired (Figure 2(d)), and the tracheal perforation was not repaired because of the difficulty and danger in the exposure of the posterior perforation. Then, we performed posterior instrumentation, stabilization, and fusion (Figure 2(e)). A tracheostomy tube was reinserted after surgery, with the cuff distal to the tracheal perforation. Primary wound healing occurred in both incisions. By the last follow-up (24 months), the patient could breathe normally, and the CT scan showed an intact trachea and a good cervical alignment and solid bone fusion (Figure 2(f)). Informed consent was obtained from each patient.

## 3. Discussion

Anterior cervical plates have increasingly been used for ACDF (anterior cervical discectomy and fusion) and ACCF to increase the stability of the cervical spine [1]. The most frequent hardware complication is a broken or loosened screw or plate, with an incidence of 2.1% [2]. Most spine surgeons are aware of the potential danger for the esophagus by displaced implants, but they ignore the possibility of tracheal injury. The esophageal perforation rate after anterior cervical spine surgery is 0.2% [2]. However, the rate of tracheal injury should be much lower than that of the esophagus due to the anatomical position of the trachea in front of the esophagus. Only two studies reported perforation of the trachea in two patients as a complication of the anterior cervical instrumentation failure (Table 1).

To our knowledge, this is the first report describing two patients with tracheal perforation after cervical spine trauma surgery. The mechanism of the erosion of an anterior cervical plate into the trachea is not well understood. In the patient described by Kuo et al., the loosened anterior cervical plate eroded into the pharyngoesophageal junction, leading to the development of a pharyngotracheal fistula at 10 years after primary ACDF. Therefore, the authors hypothesized that the chronic contact between the pharyngoesophageal junction and the adjacent cervical plate caused pressure necrosis and a reaction against a foreign body, with the subsequent erosion of the plate into the lumen [3]. Unlike the above patient, the perforation observed in our two patients occurred at an early period after surgery. A potential mechanism was hypothesized as follows. First, the anterior fixation alone could not be strong enough to maintain the reduction of the traumatic cervical spine dislocation, which could lead to an early screw loosening and backed out. The relatively sharp edge of a loosened screw or plate abutting the trachea could be responsible for the erosion. Second, both patients were subjected to tracheostomy intubation, and the tracheal posterior wall was compressed between the tube and the loosened plate. The contraction of the trachea during swallowing could accentuate the compression between the tube and the plate, which might accelerate the erosion of the trachea. Third, the iatrogenic injury of the trachea caused by endotracheal intubation might represent another risk factor. The reported incidence of airway injury after endotracheal intubations is estimated as 0.005% [5]. In case of cervical spine dislocation and soft tissue swelling, the risk of tracheal injury might increase during endotracheal intubation. Thus, all the above factors might have contributed to the tracheal perforation in our two patients.

There have been many reports about esophageal perforation after anterior cervical spine surgery [2]. Theoretically, the esophagus is more likely to have complications rather than tracheal perforation due to the anatomical characteristics. Due to the critical condition of our two patients, esophagography or esophagoscopy was unable to be performed, and CT scan showed no abnormalities such as fluid accumulation around the esophagus. During revision surgery, intraoperative adhesions made it difficult for clear exposure of the esophagus, but we found no significant esophageal injury. In case, the patients were treated with gastric tube for gastrointestinal nutrition and were with normal swallowing at discharge. Based on the above, there should be no esophageal perforation. The possible reasons may be as follows. Because of anatomic factors, trauma, or the primary cervical spine surgery, the esophagus is pushed to the left front of the vertebral body rather than directly in front, resulting in contact of implants with the trachea. As can be seen from Figure 1(d), the esophagus is in left front of the vertebral body (the white arrow indicates the esophagus, with the gastric tube inside, and the red arrow indicates the trachea, with the endotracheal tube inside), while the trachea is in contact with the implants directly in front of the vertebral body. That might explain why the trachea was perforated while the esophagus was not.

The clinical manifestations of tracheal injury can be nonspecific and may include coughing, dyspnea, laryngopharyngeal polyp, subcutaneous emphysema, pneumomediastinum, and even acute respiratory failure [3, 5, 6]. This report actually demonstrated that patients with tracheal perforation could be asymptomatic and therefore diagnosis might be delayed. Bronchoscopy is the “gold standard” in the diagnosis of tracheal injury [5]. However, CT scan was also crucial in the diagnosis and evaluation of this condition in these two patients (Figures 1(f) and 2(c)).

Tracheal perforation in most instances needs an individualized treatment based on the patient's comorbidities, clinical presentation, and anatomy [5]. According to the literature, the degree of tracheal wall injury in both our patients was level II (perforation of the posterior tracheal wall less than 2 cm), and a nonsurgical management should be considered, with the diversion of the airflow through a tracheostomy tube placed more distally [5, 7, 8]. The surgical repair of the tracheal perforation using muscle flap is reported in a previous study, but the size of the perforation was not described [4]. In our two patients, anterior implant removal, thorough debridement and irrigation, multilevel reliable posterior fracture reduction, fixation, and fusion (which could be procedures performed by one-stage surgery or multiple-stage surgeries) were necessary, and the anterior vertebral defect was either reconstructed with autologous iliac graft or left unrepaired. The endotracheal intubation should be performed with care before the revision procedure to avoid further injury of the posterior tracheal wall, and the use of a fiber-optic bronchoscopy is recommended. A tracheostomy tube should be reinserted after surgery to enhance pulmonary hygiene and the healing of the tracheal perforation.

In conclusion, this work described two patients with perforation of the trachea following anterior cervical spine surgery due to the displacement of the implants. Although very rare and challenging, this complication could be managed by posterior cervical instrumentation and fusion, anterior implant removal and debridement, and the use of a distal bypassing tracheostomy tube.

## Figures and Tables

**Table 1:** Patients with tracheal perforation after anterior cervical spine surgery.

Author	Kuo and Levine [3]	Pariyadath et al. [4]	Our cases	
Age/gender	58/F	34/F	54/F	50/M
Initial lesion	Cervical radiculopathy	Epidural abscess of the cervical spine	C7 fracture and C6 dislocation	C7 dislocation
Initial surgery	C4-C7 ACDF	Drainage of abscess and ACDF	C7 ACCF, C6-T1 fixation	C6-C7 partial ACCF, C5-T1 fixation
Time until the diagnosis of tracheal perforation	10 years	Early period after surgery	35 days	13 days
Revision surgery	Removal of the implants and reparation of the pharyngoesophageal defect	Removal of the implants and realization of the posterior fusion in two stages	Removal of the implants and realization of the posterior fixation in two stages	Removal of the implants and realization of the posterior fixation in one stage
Management of the tracheal perforation	Unrepaired, with the placement of a nasogastric tube	Reparation of the tracheal injury with a muscle flap and placement of a tracheotomy tube	Unrepaired, with the placement of a tracheotomy tube	Unrepaired, with the placement of a tracheotomy tube
Clinical outcome	Successful healing	Successful healing	Successful healing	Successful healing
Period of follow-up	3 moths	Few days	16 months	Two years

## Data Availability

We shall make data available to researchers in the related area under reasonable request. They could get the individual participant data that underlie the results reported in this article. Data can be obtained by contacting the corresponding author.
